# Supplementary material for: The mouse multi-organ proteome from infancy to adulthood
Source: Nat Commun. 2024 Jul 9;15:5752. doi: 10.1038/s41467-024-50183-6 (PMC11233712; doi:10.1038/s41467-024-50183-6)
Supplement: Supplementary file 1 — Supplementary Information [file 41467_2024_50183_MOESM1_ESM.pdf]

The mouse multi-organ proteome from infancy to adulthood

Qingwen Wang[1,2], Xinwen Ding[1,2], Zhixiao Xu[1,2], Boqian Wang[1,2], Aiting Wang[1,2], Liping Wang[1], Yi Ding[1,2], Sunfengda Song[1,2], Youming Chen[1,2], Shuang Zhang[1,2], Lai Jiang[2], Xianting Ding[1,2]*

1. State Key Laboratory of Oncogenes and Related Genes, Institute for Personalized Medicine, Shanghai Jiao Tong University, Shanghai, China
2. Department of Anesthesiology and Surgical Intensive Care Unit, Xinhua Hospital, School of Medicine and School of Biomedical Engineering, Shanghai Jiao Tong University, Shanghai, China
* Corresponding Author: Xianting Ding, E-mail: dingxianting@sjtu.edu.cn

Supplementary Figures

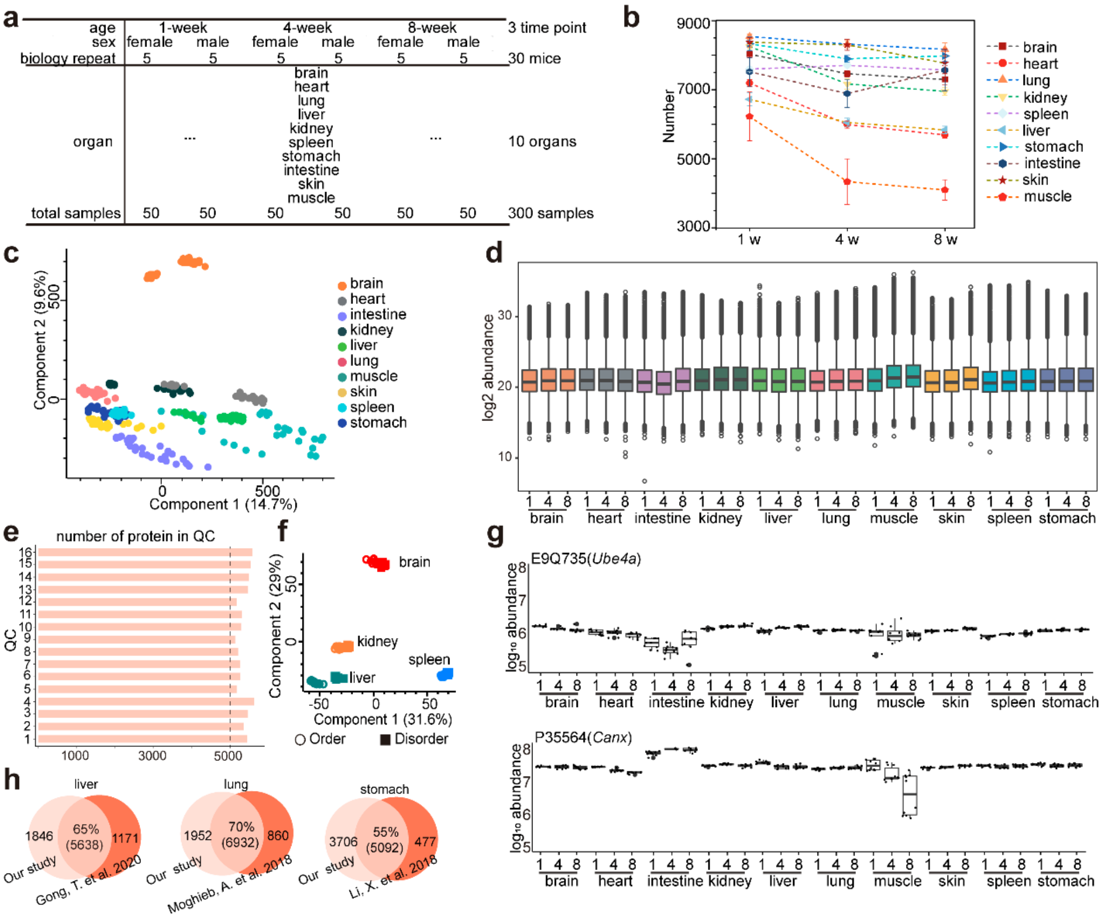

Supplementary Fig. 1 | Proteomic atlas of ten organs from infancy to adulthood mice. **a,** Summary of the sample age, organ type, number of mice, and total number of samples. **b,** Line plots depicting the alterations in the count of protein identifications across three different ages were generated for ten distinct organs. **c,** PCA analyses were performed on proteomic data from all ten organs without any filtering on samples or proteins. The samples clustered based on organ type but not by batch. **d,** Protein abundances (in log2 scale) in each age group for each organ. In the box plot, the median is represented by the center line and the box boundaries represent the first and third quartiles. **e,** The number of proteins identified by QC was stable above 5000, showing that no effect along measurement time on instrument performance was observed. **f,** PCA visualization was performed on a total of 40 samples from the brain, kidney, liver, and spleen after conducting ordered and unordered detection. **g,** Dynamic Changes in housekeeping protein expression. In the box plot, the median is represented by the center line and the box boundaries represent the first and third quartiles. In the box plot, the median is represented by the center line and the box boundaries represent the first and third quartiles. n=10 biologically independent mice per organ per age. **h,** Venn diagrams show the comparison of the protein species identified compared to the results of other single-organ studies. Source data are provided as a Source Data file.

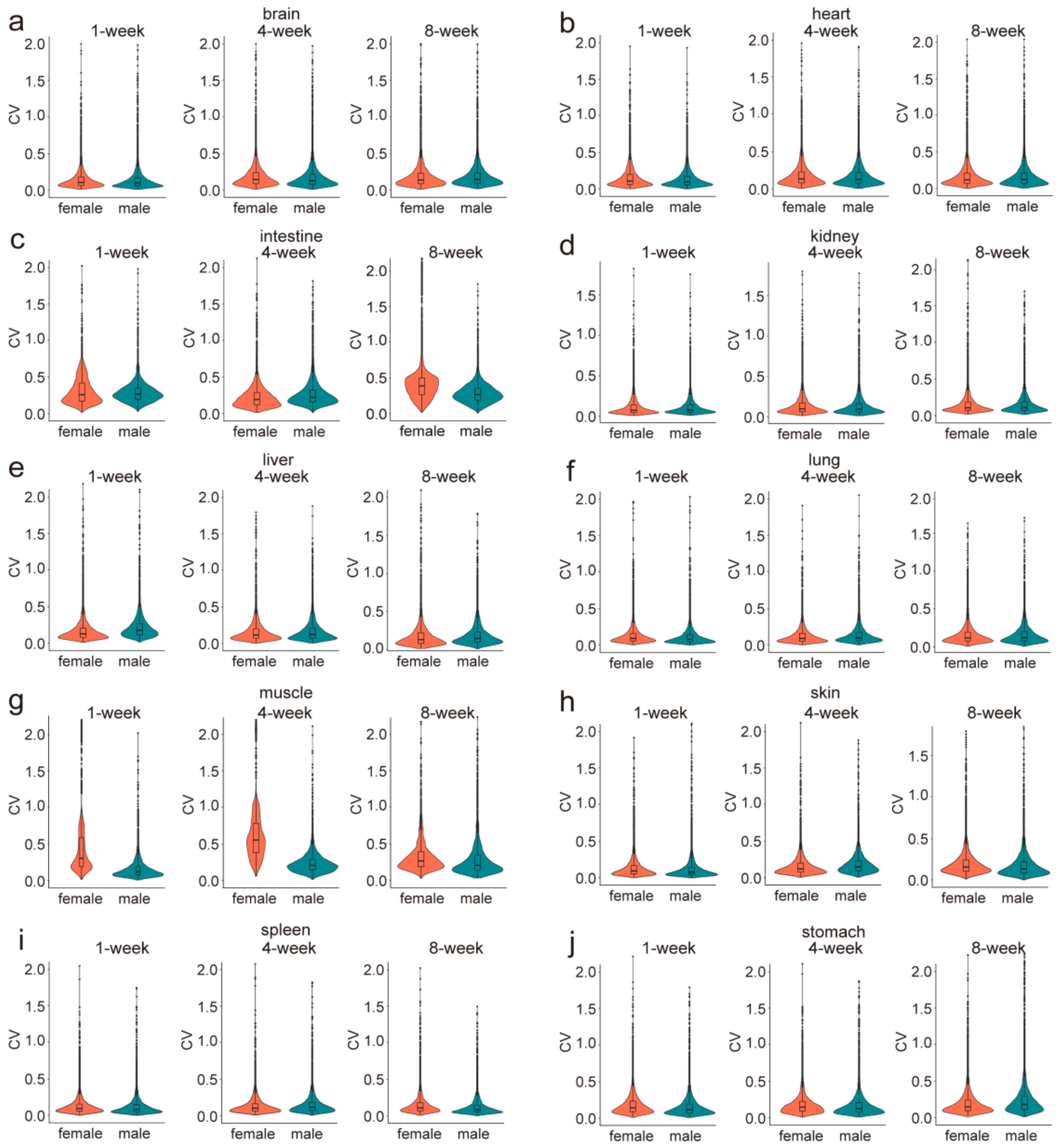

Supplementary Fig. 2 | Coefficient of variation (CV) across biological replicates. The median CV of the proteomics data was calculated by the normalized quantified proteins. **a,** brain. **b,** heart. **c,** intestine. **d,** kidney. **e,** liver. **f,** lung. **g,** muscle. **h,** skin. **i,** spleen. **j,** stomach. In the box plot, the median is represented by the center line and the box boundaries represent the first and third quartiles. n=5 biologically independent mice of each sex. Source data are provided as a Source Data file.

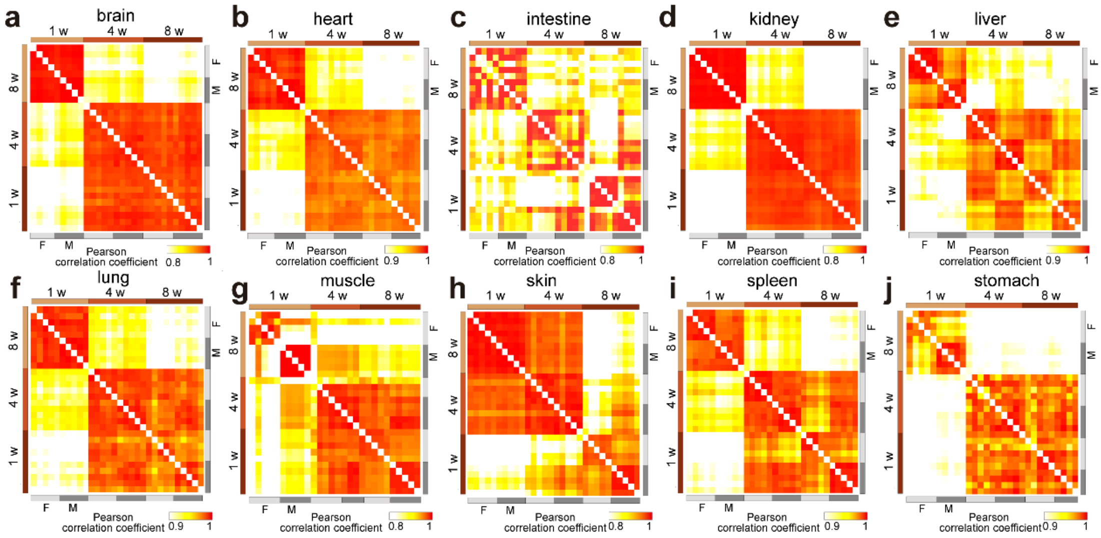

Supplementary Fig. 3 | Pearson correlation coefficient heatmaps of 10 organs. a, brain. **b,** heart. **c,** intestine. **d,** kidney. **e,** liver. **f,** lung. **g,** muscle. **h,** skin. **i,** spleen. **j,** stomach. n = 9 biologically independent samples in 1-week-brain group, n = 10 biologically independent samples in all other age-organ groups. Source data are provided as a Source Data file.

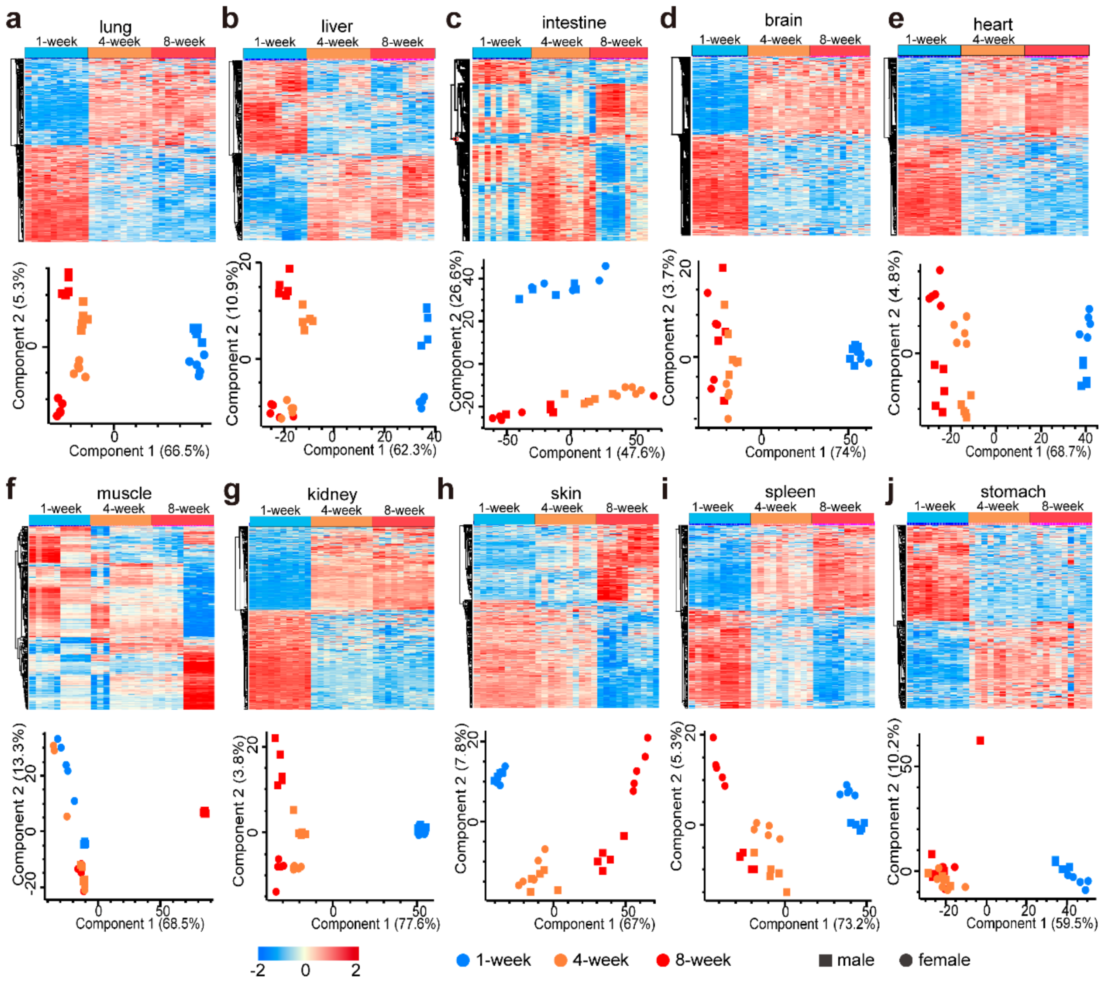

Supplementary Fig. 4 | Age-related differential proteins in each organ. a, lung. **b,** liver. **c,** intestine. **d,** brain. **e,** heart. **f,** muscle. **g,** kidney. **h,** skin. **i,** spleen. **j,** stomach. Top: heatmap of age-related differential proteins; bottom: principal component analysis (PCA) of the age-related proteomic data. n = 9 biologically independent samples in 1-week-brain group, n = 10 biologically independent samples in all other age-organ groups. Source data are provided as a Source Data file.

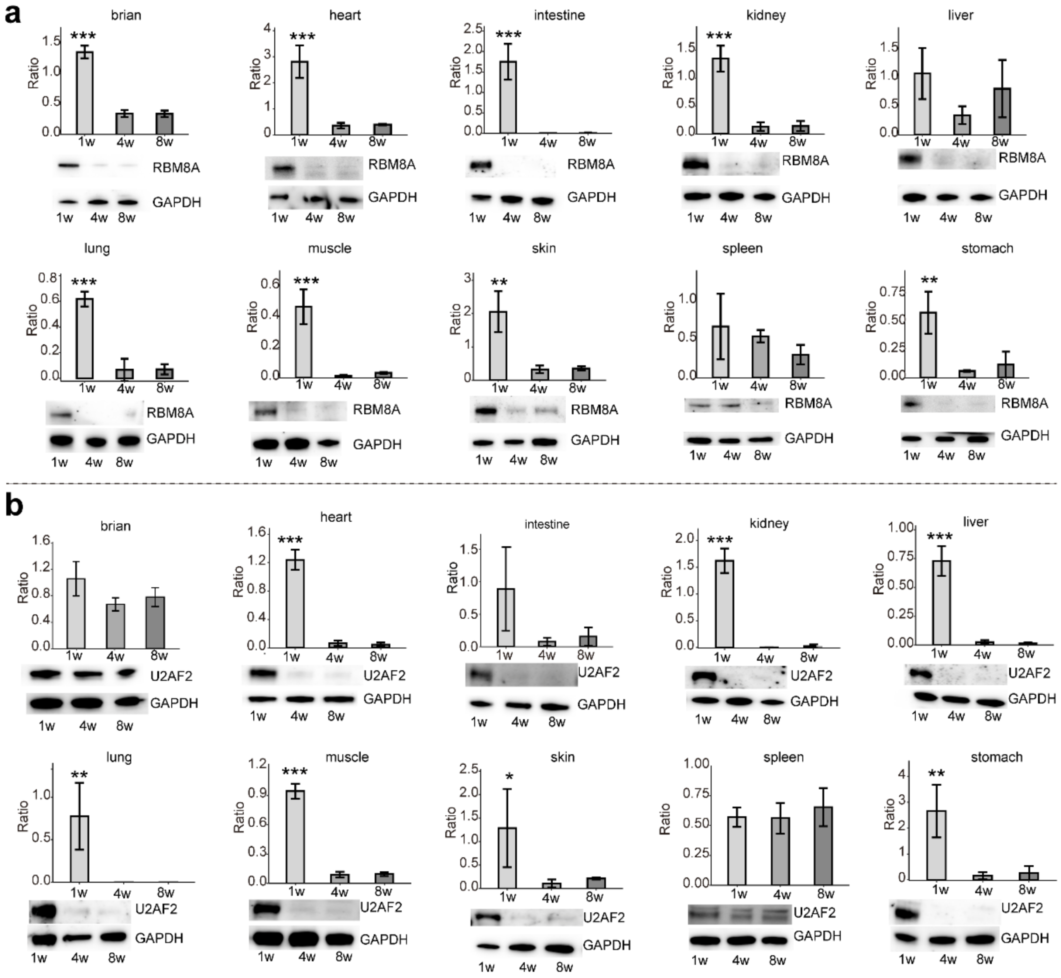

Supplementary Fig. 5 | Western blots results of spliceosome protein: RBM8A(a), and U2AF2(b). Ratio = target protein gray value: GAPDH gray value. (mean ± SD; n=3 biologically independent mice). * p-value < 0.05, ** p-value < 0.01, *** p-value < 0.001 according to a one-way ANOVA. Source data are provided as a Source Data file.

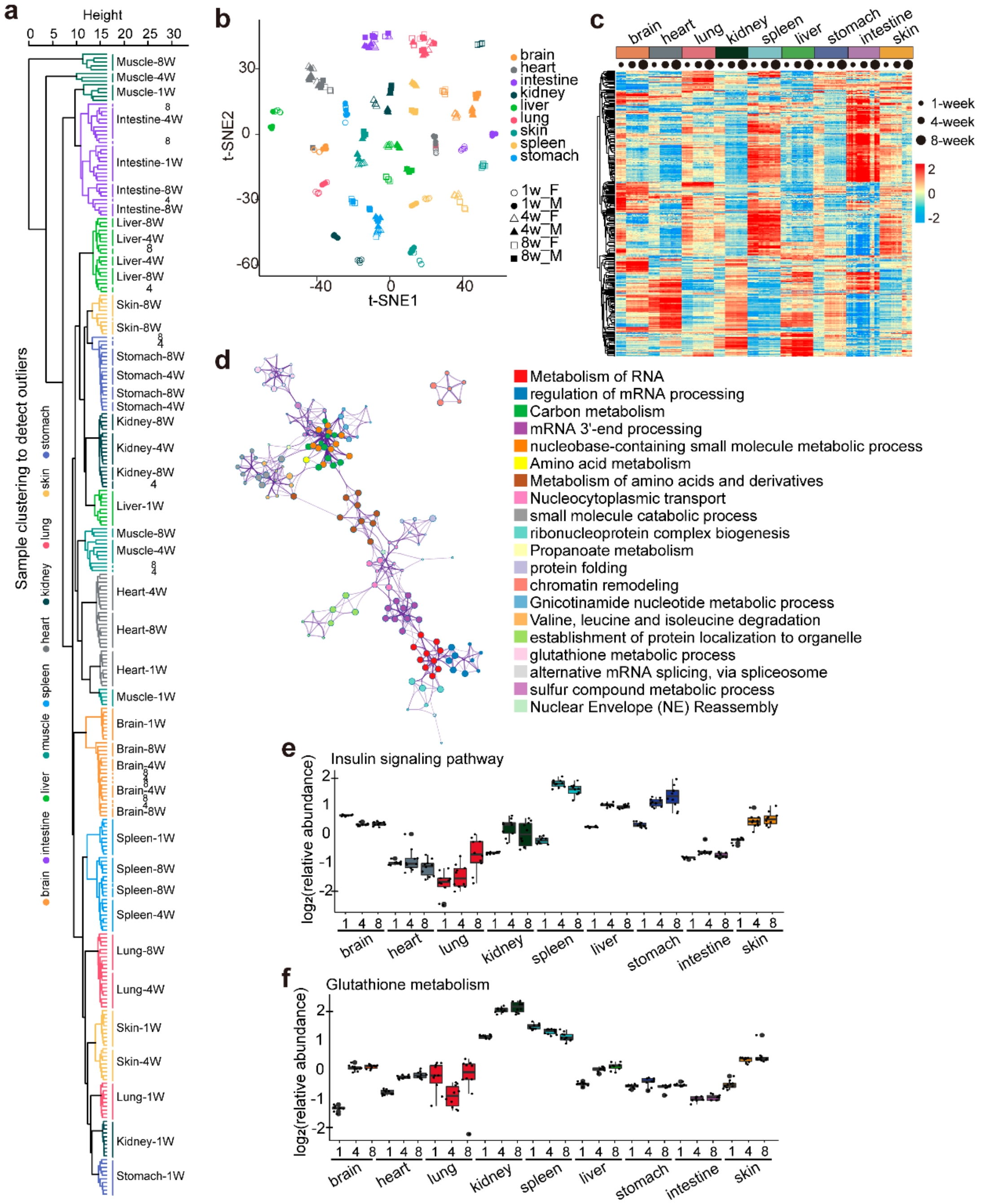

Supplementary Fig. 6 | Age-related differential proteins across 9 organs from infancy to adulthood. a, Hierarchical clustering of 299 samples. **b,** t-SNE visualization of the data from 566 DEPs co-expressed in 9 organs. **c,** Heatmap of the 566 DEPs expressed across different ages and organs. **d,** Metascape enrichment network of top 20 non-redundant enrichment clusters. Cluster annotations are shown in color code. Temporal expression patterns of proteins involved in the insulin signaling pathways (**e**) and glutathione metabolism (**f**). In the box plot, the median is represented by the center line and the box boundaries represent the first and third quartiles. In the box plot, the median is represented by the center line and the box boundaries represent the first and third quartiles. n = 9 biologically independent samples in 1-week-brain group, n = 10 biologically independent samples in all other age-organ groups. Source data are provided as a Source Data file.

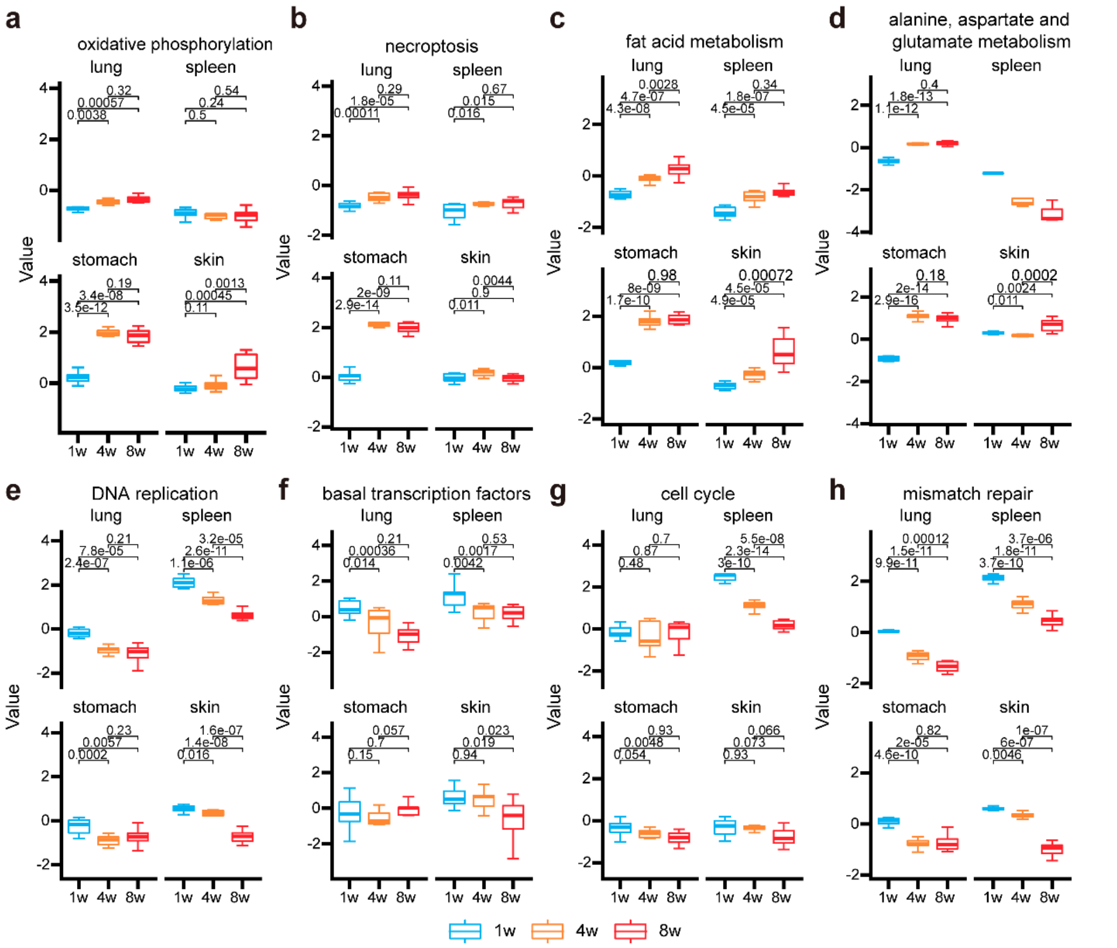

Supplementary Fig. 7 | Dynamic expression of age-related differential proteins in lung, spleen, stomach, and skin. a, Oxidative phosphorylation. **b**, Necroptosis. **c**, Fat acid metabolism. **d**, alanine, aspartate and glutamate metabolism. **e**, DNA replication. **f**. Basal transcription factors. **g**, Cell cycle. **h**, mismatch repair. In the box plot, the median is represented by the center line and the box boundaries represent the first and third quartiles. In the box plot, the median is represented by the center line and the box boundaries represent the first and third quartiles. The *p*-value is the raw result of statistical analysis used two-tailed unpaired *t*-test. n=10 biologically independent mice per organ per age. Source data are provided as a Source Data file.

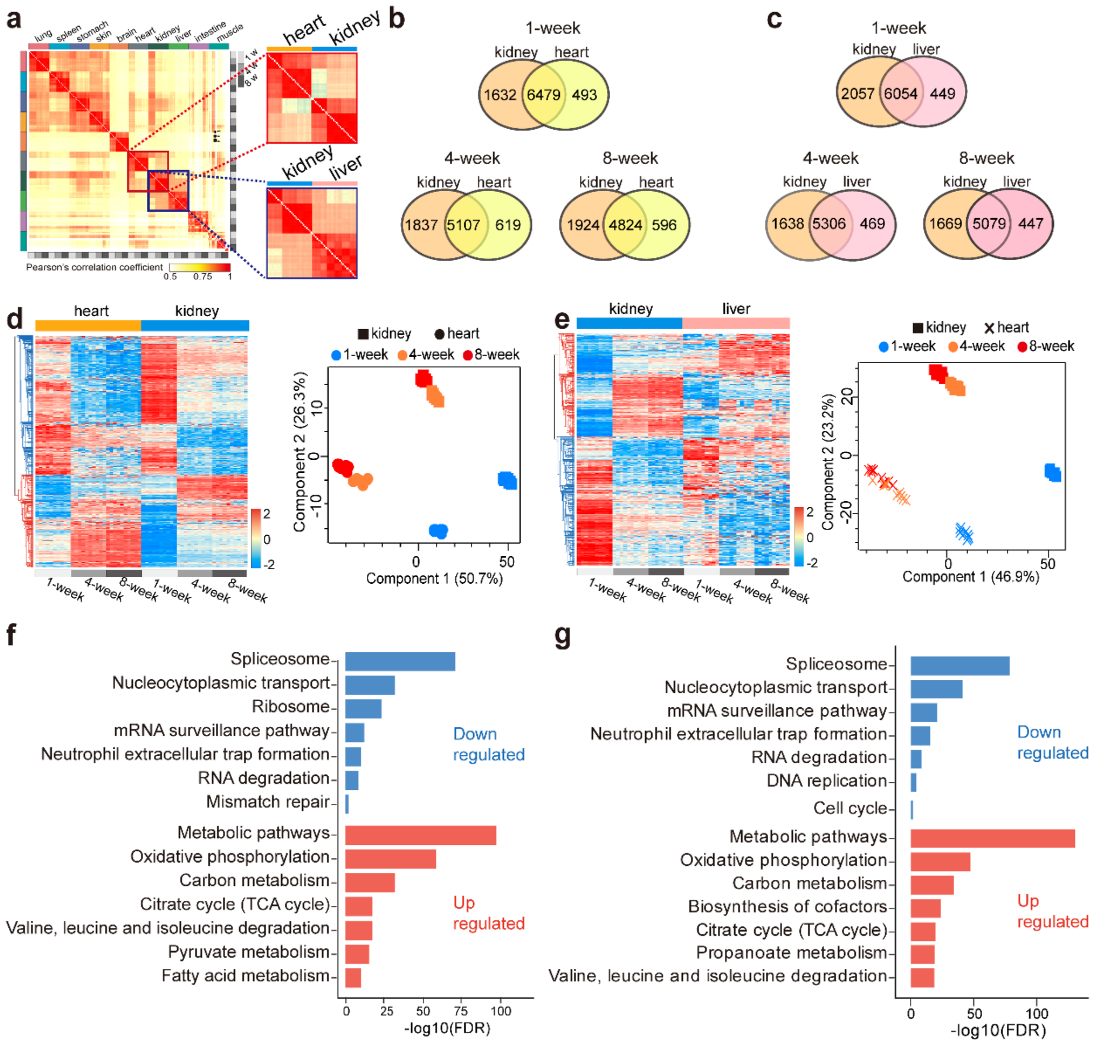

Supplementary Fig. 8 | Age-related expression of DEPs in the heart and kidney and in the kidney and liver. a, The heart and kidney, the kidney and liver are clustered together separately in **Figure 1 d** indicating that they are more interconnected to each other. **b,** Venn diagram of protein between the heart and kidney from infancy to adulthood. **c,** Venn diagram of protein between the heart and kidney from infancy to adulthood. **d,** Heatmap of the two expression modules revealed by k-means clustering in the heart and kidney (left). Principal component analysis (PCA) of the age-related DEPs in the heart and kidney (right). **e,** Heatmap of the two expression modules revealed by k-means clustering in the kidney and liver (left). Principal component analysis (PCA) of the age-related DEPs in the kidney and liver (right). **f,** Results of KEGG analysis for up-regulated proteins and down-regulated proteins in the heart and kidney separately. **g,** Results of KEGG analysis for up-regulated proteins and down-regulated proteins in the kidney and liver separately. FDR were estimated in DAVID software using Fisher's Exact test. Source data are provided as a Source Data file.

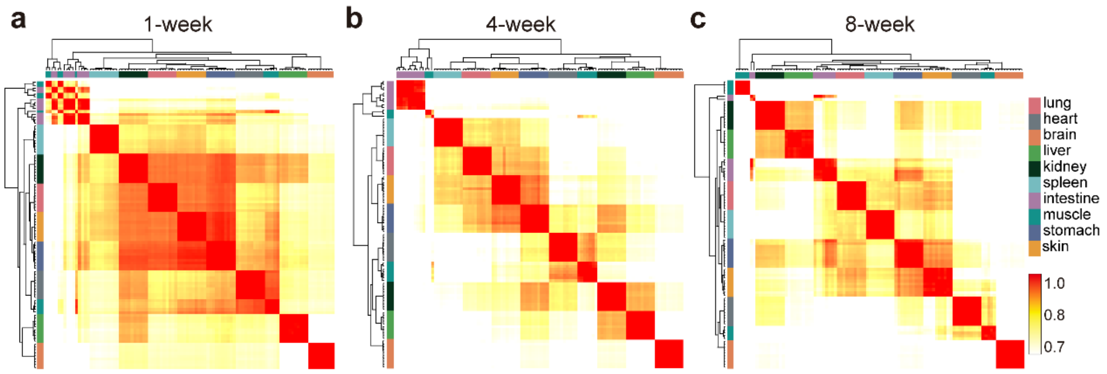

Supplementary Fig. 9 | Pearson correlation coefficient of ten organs proteomic data from infancy to adulthood. a, 1 week. **b**, 4-week. **c**, 8-week. n = 9 biologically independent samples in 1-week-brain group, n = 10 biologically independent samples in all other age-organ groups. Source data are provided as a Source Data file.

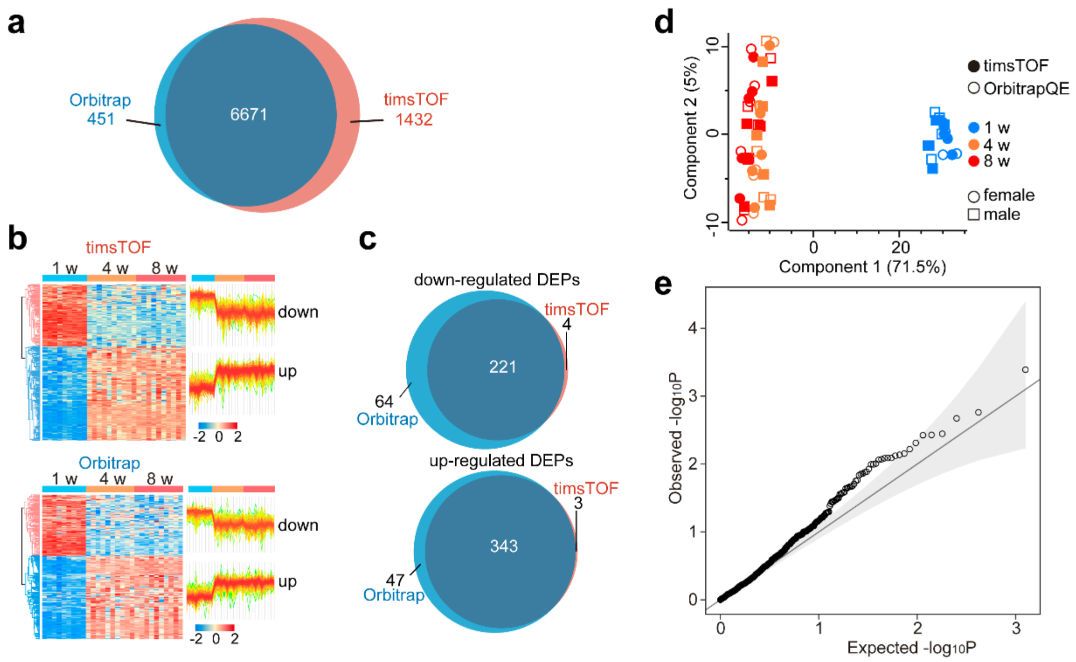

Supplementary Fig. 10 | Brain-unique age-related DEPs validation. a, Venn diagram shows brain tissue proteomic data obtained from two mass spectrometry instruments, Orbitrap QE HF-X system (Thermo Fisher Scientific) and timsTOF (Bruker). **b,** Heatmap of brain-unique age-related DEPs by semi-supervised hierarchical clustering. Top: data from timsTOF (Bruker); Bottom: data from Orbitrap (Thermo Fisher Scientific). The protein expression trends fall into the down-regulated module and the up-regulated module. **c,** Venn diagram shows down-regulated DEPs data (top) and up-regulated protein data （bottom）obtained from two mass spectrometers. **d,** PCA analyses of proteomic data from Obritrap QE and timsTOF LC-MS platform. **e,** Q–Q plots to compare the *p* values reported of the data detected by the two mass spectrometers with the normalized rank *p* values that represent the uniform distribution. The data points exhibit an evenly distributed pattern along the diagonal, indicating strong reproducibility of the detected data across both platforms. n = 9 biologically independent samples. Source data are provided as a Source Data file.

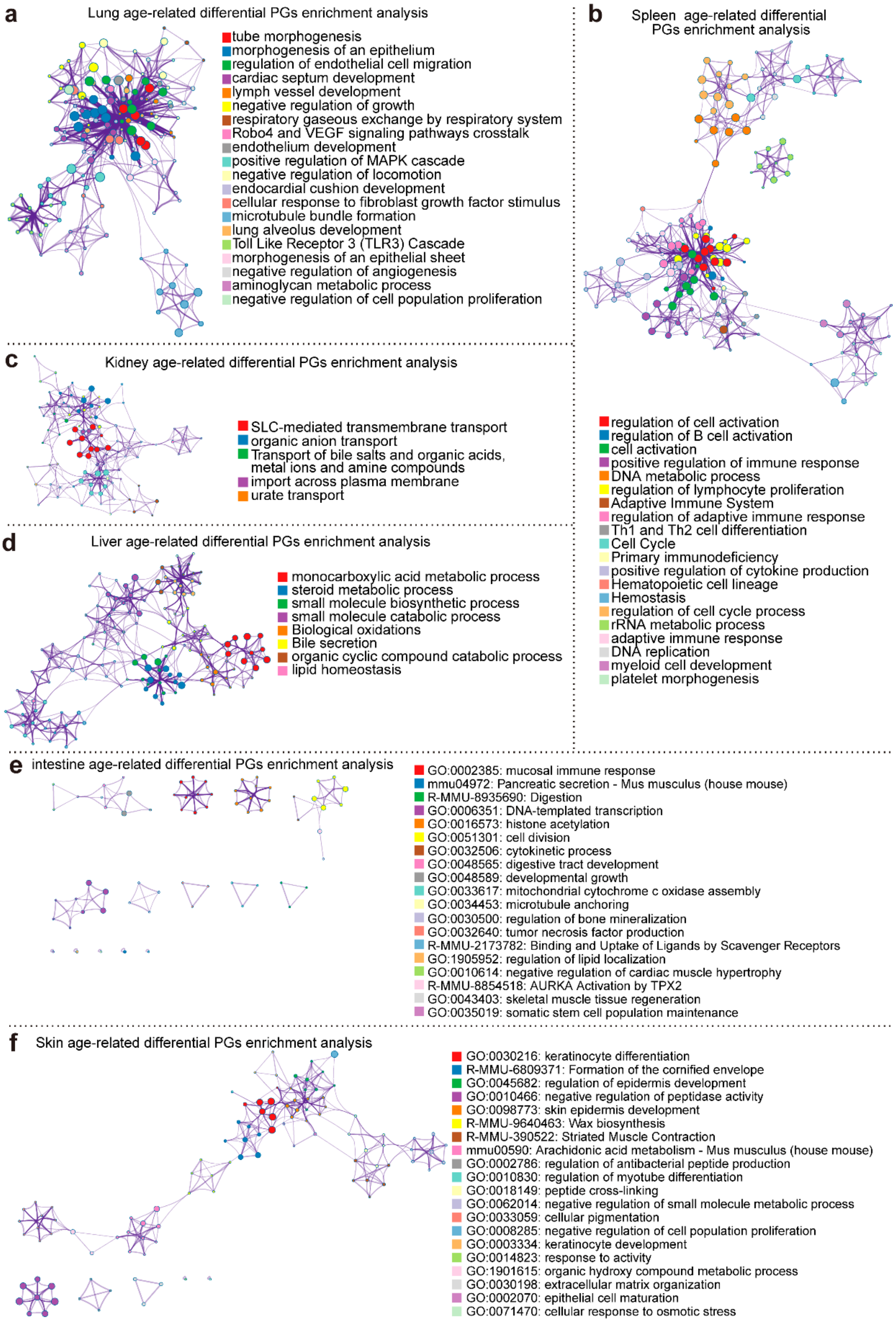

Supplementary Fig. 11 | Top non-redundant enrichment clusters of each organ-unique age-related DEPs. a, Lung age-related differential PGs enrichment analysis. **b,** Spleen age-related differential PGs enrichment analysis. **c,** Kidney age-related differential PGs enrichment analysis. **d,** Liver age-related differential PGs enrichment analysis. **e,** Intestine age-related differential PGs enrichment analysis. **f,** Skin age-related differential

PGs enrichment analysis. Source data are provided as a Source Data file.

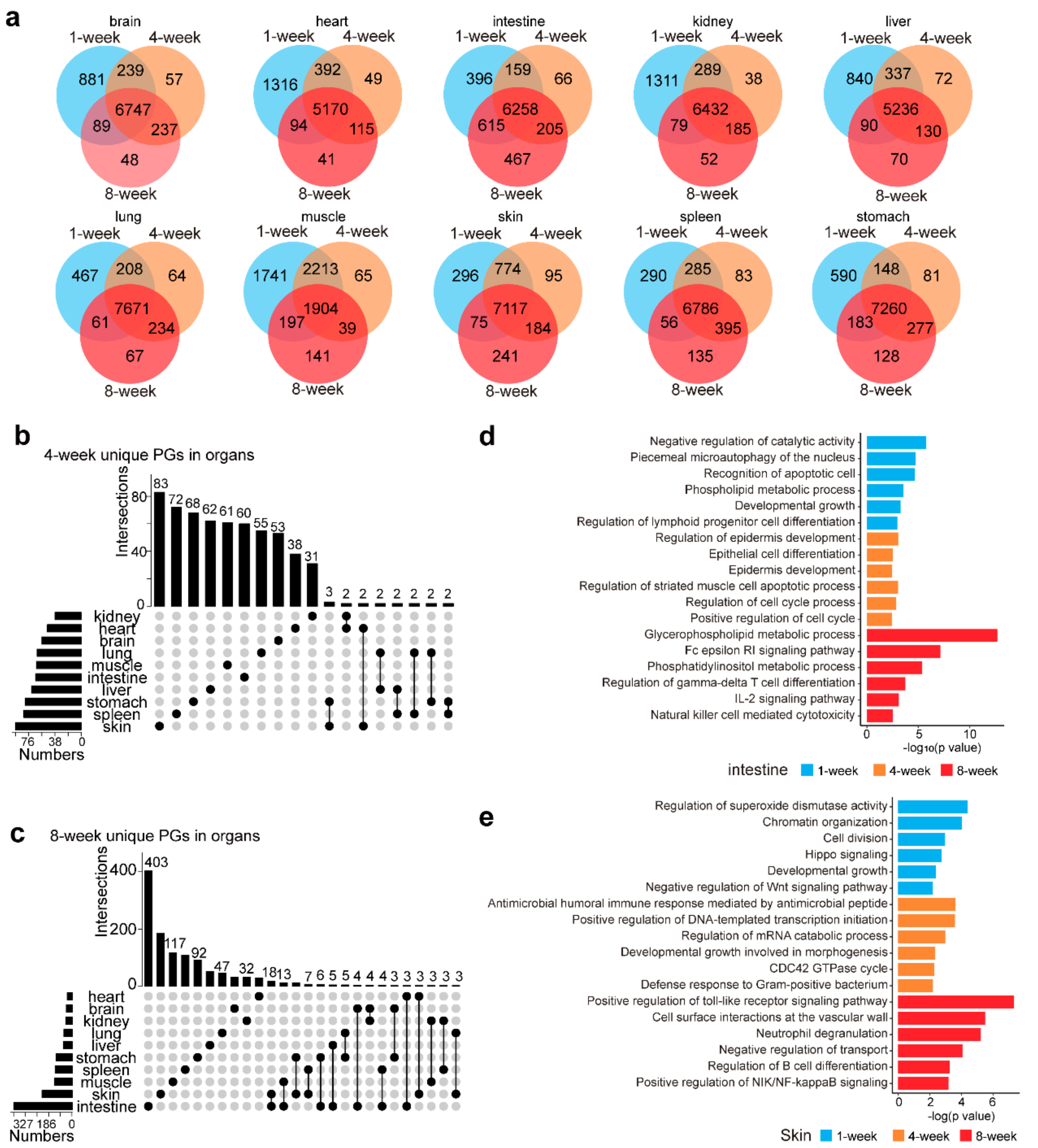

Supplementary Fig. 12 | Age-unique proteins in 10 organs. a. Venn diagram of differential age in each organ. The number of 4-week unique proteins (**b**) and 8-week unique proteins(**c**) in 10 organs. The number of proteins in each organ that are only expressed in a single organ (bars, left). Protein counts (top, bars) are found in each combination of organs or a single organ (bottom, black dots). GO enrichment analysis of age-unique proteins in intestine (**d**) and skin (**e**). *p*-value were estimated in DAVID software using Fisher's Exact test. Source data are provided as a Source Data file.

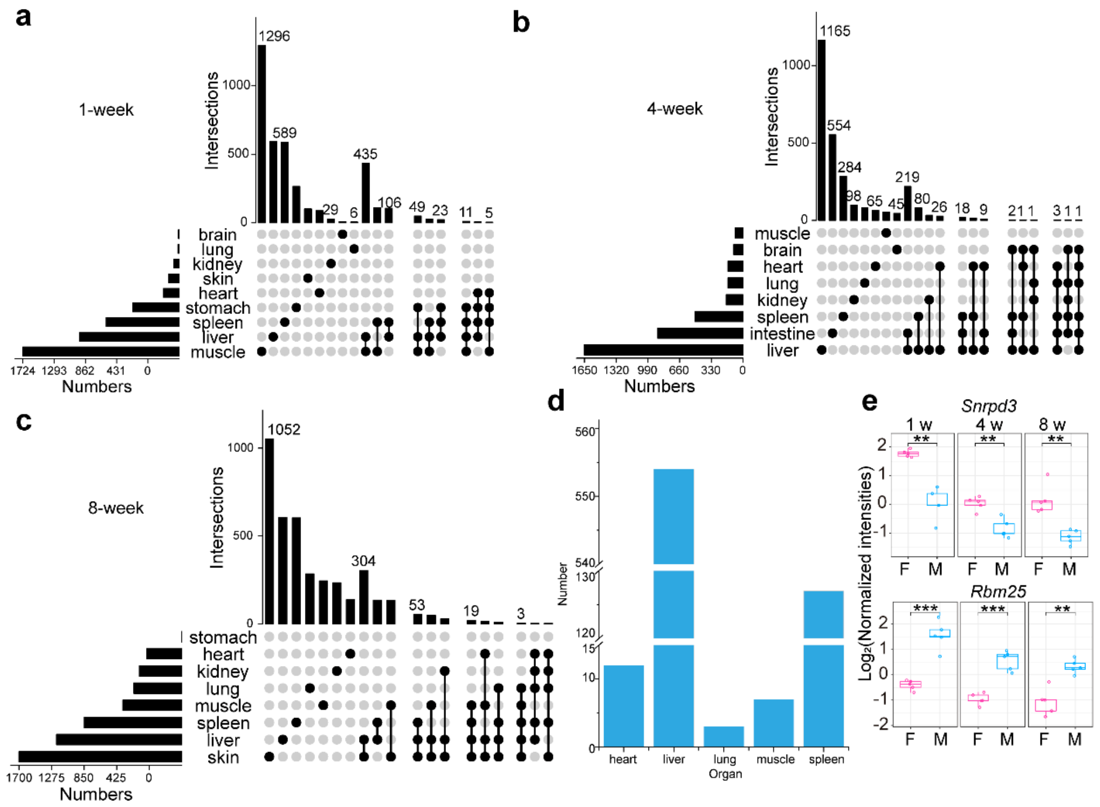

Supplementary Fig. 13 | Sex-related DEPs. Number of sex-related differential proteins (DEPs) in various organs. The number of proteins in each organ that are only expressed in 1-week organ (**a**), 4-week organ (**b**), and 8-week organ (**c**). Protein counts (top, bars) are found in each combination of organs or a single organ (bottom, black dots). **d.** The bar chart shows the number of sex-related DEPs present at all three age points. **e**. Boxplot showing the splicing proteins *Snrpd3* and *Rbm25* expressed in different sexes. Statistical analysis used two-tailed unpaired *t*-test (** *p*-value < 0.01, *** *p*-value < 0.001). In the box plot, the median is represented by the center line and the box boundaries represent the first and third quartiles. n=5 biologically independent mice of each sex. Source data are provided as a Source Data file.
