# Supplementary material for: The mouse multi-organ proteome from infancy to adulthood
Source: Nat Commun. 2024 Jul 9;15:5752. doi: 10.1038/s41467-024-50183-6 (PMC11233712; doi:10.1038/s41467-024-50183-6)
Supplement: Supplementary file 3 — Description of Additional Supplementary Files [file 41467_2024_50183_MOESM3_ESM.pdf]

Description of Additional Supplementary Files

Supplementary Data Legends:

Supplementary Data 1: ten organs proteome atlas from infancy to adulthood.

Supplementary Data 2: log10 transformed quanΘtaΘve data.

Supplementary Data 3: each organ Pearson correlaΘon data.

Supplementary Data 4: Comparison of data between sequenΘal sampling and random sampling.

Supplementary Data 5: CV in every biological replicates.

Supplementary Data 6: ten organs ANOVA analysis results.

Supplementary Data 7: Results of KEGG analysis of age-related differenΘal proteins per organ.

Supplementary Data 8: Age-related DEPs across ten organs from infancy to adulthood.

Supplementary Data 9: Age-related DEPs across nine organs from infancy to adulthood.

Supplementary Data 10: lung spleen stomach skin co-expressed data analysis.

Supplementary Data 11: heart_kidney and kidney_liver coexpresed data analysis.

Supplementary Data 12: Pearson CorrelaΘon for ten organs at 1-week, 4-week and 8-week respecΘvely.

Supplementary Data 13: ten organs comparaΘve analysis of organ-unique agerelated differenΘal protein enrichment analysis results.

Supplementary Data 14: brain_unique agerelated differenΘal proteins analysis results.

Supplementary Data 15: Tims TOF mass spectrometry validated data.

Supplementary Data 16: ageunique proteins in ten organs.

Supplementary Data 17: Metascape results of sex DEPs.
