# Supplementary material for: The mouse multi-organ proteome from infancy to adulthood
Source: Nat Commun. 2024 Jul 9;15:5752. doi: 10.1038/s41467-024-50183-6 (PMC11233712; doi:10.1038/s41467-024-50183-6)
Supplement: Supplementary file 21 — Reporting Summary [file 41467_2024_50183_MOESM21_ESM.pdf]

nature portfolio

Corresponding author(s): Xianting Ding

Last updated by author(s): May 2, 2024

Reporting Summary

Nature Portfolio wishes to improve the reproducibility of the work that we publish. This form provides structure for consistency and transparency in reporting. For further information on Nature Portfolio policies, see our Editorial Policies and the Editorial Policy Checklist.

Statistics

For all statistical analyses, confirm that the following items are present in the figure legend, table legend, main text, or Methods section.

n/a	Confirmed	
☐	☒	The exact sample size (*n*) for each experimental group/condition, given as a discrete number and unit of measurement
☐	☒	A statement on whether measurements were taken from distinct samples or whether the same sample was measured repeatedly
☐	☒	The statistical test(s) used AND whether they are one- or two-sided<br>*Only common tests should be described solely by name; describe more complex techniques in the Methods section.*
☐	☒	A description of all covariates tested
☐	☒	A description of any assumptions or corrections, such as tests of normality and adjustment for multiple comparisons
☐	☒	A full description of the statistical parameters including central tendency (e.g. means) or other basic estimates (e.g. regression coefficient) AND variation (e.g. standard deviation) or associated estimates of uncertainty (e.g. confidence intervals)
☐	☒	For null hypothesis testing, the test statistic (e.g. *F*, *t*, *r*) with confidence intervals, effect sizes, degrees of freedom and *P* value noted<br>*Give P values as exact values whenever suitable.*
☒	☐	For Bayesian analysis, information on the choice of priors and Markov chain Monte Carlo settings
☐	☒	For hierarchical and complex designs, identification of the appropriate level for tests and full reporting of outcomes
☐	☒	Estimates of effect sizes (e.g. Cohen's *d*, Pearson's *r*), indicating how they were calculated

Our web collection on statistics for biologists contains articles on many of the points above.

Software and code

Policy information about availability of computer code

Data collection	All mass spectrum data were collected with EASY nLC 1200 coupled with QE HF-X system (Thermo Fisher Scientific). The mass spectrometry validation experiment collected with timsTOF flex.
Data analysis	MSConvertGUI (part of proteoWizard 3.0.22068) was used to convert Raw data to the .mzML format. All DIA raw files were analyzed using the DIA_NN-1.8.1. Proteome Discoverer 2.4.0.305 (Thermo Fisher Scientific) was used for DDA raw data protein detection. Perseus software (v1.6.15.0), R (v4.2.0), Origin (v2022b), and the "Wu Kong" platform were used for biometric analysis and visualization. Gene ontology (GO), Kyoto Encyclopedia of Genes and Genomes (KEGG) terms functional enrichment analysis and annotation were performed using the web-based tool DAVID (https://david.ncifcrf.gov/) and Metascape (http://metascape.org.).

For manuscripts utilizing custom algorithms or software that are central to the research but not yet described in published literature, software must be made available to editors and reviewers. We strongly encourage code deposition in a community repository (e.g. GitHub). See the Nature Portfolio guidelines for submitting code & software for further information.

Data

Policy information about availability of data

All manuscripts must include a data availability statement. This statement should provide the following information, where applicable:

- Accession codes, unique identifiers, or web links for publicly available datasets
- A description of any restrictions on data availability
- For clinical datasets or third party data, please ensure that the statement adheres to our policy

The mass spectrometry proteomics data and searching output data have been deposited to the ProteomeXchange Consortium via the PRIDE partner repository with the dataset PXD041400. A reporting summary for this Article is available as a Supplementary Information file. Source data are provided with this paper.

Research involving human participants, their data, or biological material

Policy information about studies with human participants or human data. See also policy information about sex, gender (identity/presentation), and sexual orientation and race, ethnicity and racism.

Reporting on sex and gender	none
Reporting on race, ethnicity, or other socially relevant groupings	none
Population characteristics	none
Recruitment	none
Ethics oversight	none

Note that full information on the approval of the study protocol must also be provided in the manuscript.

Field-specific reporting

Please select the one below that is the best fit for your research. If you are not sure, read the appropriate sections before making your selection.

☒ Life sciences ☐ Behavioural & social sciences ☐ Ecological, evolutionary & environmental sciences

For a reference copy of the document with all sections, see nature.com/documents/nr-reporting-summary-flat.pdf

Life sciences study design

All studies must disclose on these points even when the disclosure is negative.

Sample size	No statistical tests were used to predetermine the sample size. The sample size was determined based on the experience of previous work as well as literature from relevant studies.
Data exclusions	Proteins with at least four samples containing valid data out of the five biological replicates were retained.<br>Protein groups with less than 4 valid values in 5 biological replicates were exclusion.<br>One brain sample at 1-week was removed due to abnormal mass spectrogram acquisition, and 299 mass spectra were used for data analysis.
Replication	All data presented are biological replicates unless otherwise stated in the figure legends.
Randomization	Animals were randomly assigned to different experimental groups based on sex and age.
Blinding	We did not implement blinding in our study.

Reporting for specific materials, systems and methods

We require information from authors about some types of materials, experimental systems and methods used in many studies. Here, indicate whether each material, system or method listed is relevant to your study. If you are not sure if a list item applies to your research, read the appropriate section before selecting a response.

Materials & experimental systems

n/a	Involved in the study	
☐	☒	Antibodies
☒	☐	Eukaryotic cell lines
☒	☐	Palaeontology and archaeology
☐	☒	Animals and other organisms
☒	☐	Clinical data
☒	☐	Dual use research of concern
☒	☐	Plants

Methods

n/a	Involved in the study	
☒	☐	ChIP-seq
☒	☐	Flow cytometry
☒	☐	MRI-based neuroimaging

Antibodies

Antibodies used	mouse anti-RBM8A, proteintech, Cat. 67541-1-Ig (1:5000)<br>mouse anti-U2AF2, proteintech, Cat. 68166-1-Ig (1:5000)<br>mouse anti-GAPDH, proteintech, Cat. 60004-1-Ig (1:10000)<br>HRP-conjugated affinipure Goat anti-Mouse IgG(H+L), proteintech, Cat. SA00001-1 (1:2000)
Validation	All antibodies used in this study are from commercial suppliers that have verified the specificity of the antibodies.<br>RBM8A: https://www.ptglab.com/products/RBM8A,Y14-Antibody-67541-1-Ig.htm<br>U2AF2: https://www.ptglab.com/products/U2AF2-Antibody-68166-1-Ig.htm<br>GAPDH: https://www.ptglab.com/products/GAPDH-Antibody-60004-1-Ig.htm<br>HRP-conjugated affinipure Goat anti-Mouse IgG(H+L): https://www.ptglab.com/products/HRP-conjugated-Affinipure-Goat-Anti-Mouse-IgG-H-L-secondary-antibody.htm

Animals and other research organisms

Policy information about studies involving animals; ARRIVE guidelines recommended for reporting animal research, and Sex and Gender in Research

Laboratory animals	C57BL/6J, males (n=15, 5 at the ages of 1-week, 4-week,, and 8-week each); females(n=15, 5 at the ages of 1-week, 4-week, and 8-week each). Mice were randomly assigned in different age groups of 4-5 mice per cage according to sex and housed in a light-dark cycle of 12:12-hr, temperature of 20-26°C, humidity of 40-60%, and provided with water and standard rodent chow.
Wild animals	This study did not involve wild animals.
Reporting on sex	Both male and female mice were used in this study.
Field-collected samples	This study did not involve field-collected samples.
Ethics oversight	All experimental procedures were approved by the Institutional Biomedical Research Ethics Committee at the Institutional Animal Care and Use Committee (IACUC) of Shanghai Jiao Tong University, Shanghai, China.

Note that full information on the approval of the study protocol must also be provided in the manuscript.

Plants

Seed stocks	Not relevant.
Novel plant genotypes	Not relevant.
Authentication	Not relevant.
